# Mining Prognostic Biomarkers of Thyroid Cancer Patients Based on the Immune-Related Genes and Development of a Reliable Prognostic Risk Model

**DOI:** 10.1155/2023/6503476

**Published:** 2023-07-31

**Authors:** Hongjun Fei, Xu Han, Yanlin Wang, Shuyuan Li

**Affiliations:** Department of Reproductive Genetics, International Peace Maternity and Child Health Hospital, Shanghai Key Laboratory of Embryo Original Diseases, Shanghai Municipal Key Clinical Specialty, Shanghai Jiao Tong University School of Medicine, Shanghai 200030, China

## Abstract

**Purpose:**

Tumor immunity serves an essential role in the occurrence and development of thyroid cancer (THCA). The aim of this study is to establish an immune-related prognostic model for THCA patients by using immune-related genes (IRGs).

**Methods:**

Wilcox test was used to screen the differentially expressed immune-related genes (DEIRGs) in THCA and normal tissues, then the DEIRGs related to prognosis were identified using univariate Cox regression analysis. According to The Cancer Genome Atlas (TCGA) cohort, we developed a least absolute shrinkage and selection operator (LASSO) regression prognostic model and performed validation analyses regard to the predictive value of the model in internal (TCGA) and external (International Cancer Genome Consortium) cohorts respectively. Finally, we analyzed the correlation among the prognostic model, clinical variables, and immune cell infiltration.

**Results:**

Eighty-two of 2,498 IRGs were differentially expressed between THCA and normal tissues, and 18 of them were related to prognosis. LASSO Cox regression analysis identified seven DEIRGs with the greatest prognostic value to construct the prognostic model. The risk model showed high predictive value for the survival of THCA in two independent cohorts. The risk score according to the risk model was positively associated with poor survival and the infiltration levels of immune cells, it can evaluate the prognosis of THCA patients independent of any other clinicopathologic feature. The prognostic value and genetic alternations of seven risk genes were evaluated separately.

**Conclusion:**

Our study established and verified a dependable prognostic model associated with immune for THCA, both the identified IRGs and immune-related risk model were clinically significant, which is conducive to promoting individualized immunotherapy against THCA.

## 1. Introduction

The incidence of thyroid cancer (THCA) has shown a rapid increase over the last decade [1–3]. The American Cancer Society has projected more than 44,280 new cases of THCA and more than 2,200 associated deaths in the United States for 2021 [4]. The eighth edition of the American Joint Committee on Cancer tumor, node, metastasis (TNM) classification system listed a few factors associated with the prognosis of THCA patients, such as age, tumor size, presence of distant metastatic, presence of gross extrathyroidal extension, and so on [5, 6]. However, the staging model of THCA is still updating since the accumulation of survival data and a greater understanding of tumor behavior and clinical outcomes. In this study, we attempt to refine the prognostic risk model of THCA by incorporating molecular prognostic markers into the prognostic system to enhance the prognostic accuracy of clinical outcomes prediction and benefit to targeted therapy.

The immune system plays a key role in THCA prevention, initiation, and development [7]. The main strategies for tumor escape are the development of an immunosuppressive environment, increased resistance, and immune recognition [8]. Many studies proved that THCA cells can escape the immune response by boosting an immunosuppressive microenvironment [9–11]. To accurately stratify THCA patients according to the results of prognosis evaluation and improve management, besides histological classification, other parameters should be considered such as immune molecular features. Therefore, exploring the immune-related prognostic biomarkers of THCA is instrumental to identify new molecular targets, predicting the prognosis, implementing personalized immune precision therapy, and survival rate improvement.

It has been scientifically proven that immune-related genes (IRGs) played key roles in the systemic immune response. Our research aims to study the possible clinical availability of IRGs that in the tumor microenvironment to predict THCA prognosis, as well as their potential value as molecular biomarkers for targeted therapy. We established an IRG-based prognostic risk model by profiling an IRG dataset in the TCGA database. Subsequently, internal and external validation of the prognostic model was conducted to evaluate the feasibility of the model, and the association between the prognostic model with clinical feature and the immune status of THCA patients were further studied. In our study, the promising results can offer information for prognosis evaluation and personalized immunotherapy against THCA.

## 2. Materials and Methods

### 2.1. Data Collection and Preprocessing

We obtained the information on 2,498 IRGs from the ImmPort database (https://www.ImmPort.org/home). The transcript profiling FPKM data and the corresponding clinicopathologic information of 487 THCA patients and 58 nontumor tissues were downloaded from The Cancer Genome Atlas (TCGA, https://portal.gdc.cancer.gov) database. Microarray data concerning transcriptome profiling data and survival information of 495 THCA patients were obtained from the International Cancer Genome Consortium (ICGC) database (https://dcc.icgc.org/) for verification of the risk model.

### 2.2. Identification and Functional Enrichment Analysis of DEIRGs

We performed the differential analysis with the R package “limma” according to the expression data of the cohort from TCGA. The Wilcoxon Rank Sum test was applied to identify differentially expressed IRGs (DEIRGs) in THCA and control tissues with threshold values of false discovery rate <0.05 and |log2FoldChange| (|log2FC|) >2. We performed Kyoto Encyclopedia of Genes and Genomes pathway enrichment analysis and Gene Ontology functional annotations to make use of “clusterProfiler” R package on all DEIRGs. We also analyzed the protein–protein interaction (PPI) for all DEIRGs using the STRING database (https://string-db.org/).

### 2.3. Construction of DEIRG-Based Prognostic Risk Model with TCGA Cohort

THCA patients from the TCGA cohort were adopted as a training set to construct the prognostic model. All DEIRGs were evaluated using a univariate Cox regression model for individual risk factors affecting the survival status of THCA patients in the TCGA training group, *p* < 0.05 is the threshold. The prognostic DEIRGs identified through the previous step of univariate Cox analysis were included in the following step of constructing the least absolute shrinkage and selection operator (LASSO) Cox regression model to exclude DEIRGs that may be highly correlated with other DEIRGs. The risk score model was established based on the expression values of screened independent prognostic DEIRGs and the regression coefficient (*β*) from LASSO regression analysis. The risk score = $\sum_{i=1,2,\ldots ,n}\text{regression coefficient }\left(\text{genei}\right)\times \text{expression value of}\left(\text{genei}\right)$. The risk score is an evaluation method for the prognosis of THCA patients. Patients were stratified into two groups according to the critical value (median risk score). Patients in the low-risk score group were evaluated and had a better prognosis than the high-risk group.

We followed the methods in the paper described by Fei et al. [12] and Huo et al. [13].

### 2.4. Assessment of Prognostic Risk Model

The value of the immune-related risk model as an independent indicator for prognosis was validated in the training set of the TCGA cohort, and the independent cohort from the ICGC database separately. Kaplan–Meier survival curves of patients in different risk groups were drawn by “survminer” R package. The log-rank test was adopted to compare survival differences between the two risk groups. To estimate the predictive power of the risk score model, the receiver operating characteristic (ROC) curves were plotted using “survivalROC” R package. The area under the curve (AUC) value of the ROC curve which ranged from 0.5 to 1.0 is a discrimination criterion for the accuracy of the risk model, they are positively related.

### 2.5. Establishment of the Nomograms for THCA Survival Prediction

We further constructed a nomogram based on seven independent prognostic DEIRGs to predict survival rates of patients at 1, 3, and 5 years through the R package of “rms”.

### 2.6. Independent and Correlation Analyses between the Prognostic Model and Clinical Features

To evaluate whether the immune-related prognostic model could be an independent prognostic factor that does not rely on other clinicopathological variables (including age, gender, pathological stage, and T classification), risk score, and all clinicopathologic features were assessed through univariate and multivariate Cox analysis in the training group patients and test group patients separately. The risk score and all clinicopathologic features were used as the independent variables, overall survival (OS) as the dependent variable, hazard ratio (HR), 95% confidence interval, and *p*-value were calculated. We also assessed the correlation between the prognostic model and clinicopathologic features.

### 2.7. Correlation Analysis between Prognostic Risk Model and Immune Cells Infiltration

The immune infiltrate levels of THCA patients were derived from the TIMER database (Tumor Immune Estimation Resource algorithm, https://cistrome.shinyapps.io/timer/) and the correlation between the prognostic model and six tumor-infiltrating immune cells subsets (CD4+ T cells, B cells, dendritic cells, CD8+ T cells, neutrophils, and macrophages) were analyzed in R.

### 2.8. Evaluation of the Prognostic Value of Seven Risk Genes in THCA Patients

To clarify the function of seven risk genes in THCA, we separately evaluated the association between the expression of the seven prognostic DEARGs and OS in THCA patients using the analysis tool called GEPIA (http://gepia.cancer-pku.cn/index.html) which contains the clinical data from genotype-tissue expression and TCGA databases. We used cBioPortal (http://www.cbioportal.org) to detect the genetic alterations such as amplification, deep deletion, and various mutations of seven risk genes in THCA patients; simultaneously, the corresponding message of the tumor mutation burden (TMB) and the protein abundance data in THCA patients were also exhibited. The clinical relevance between the expression of the seven genes and their methylation status were also analyzed using the cBioPortal browser.

Moreover, a flowchart of data collection and risk model construction and verification were shown in Figure S1.

## 3. Results

### 3.1. Identification of DEIRGs

**Figure 1:**
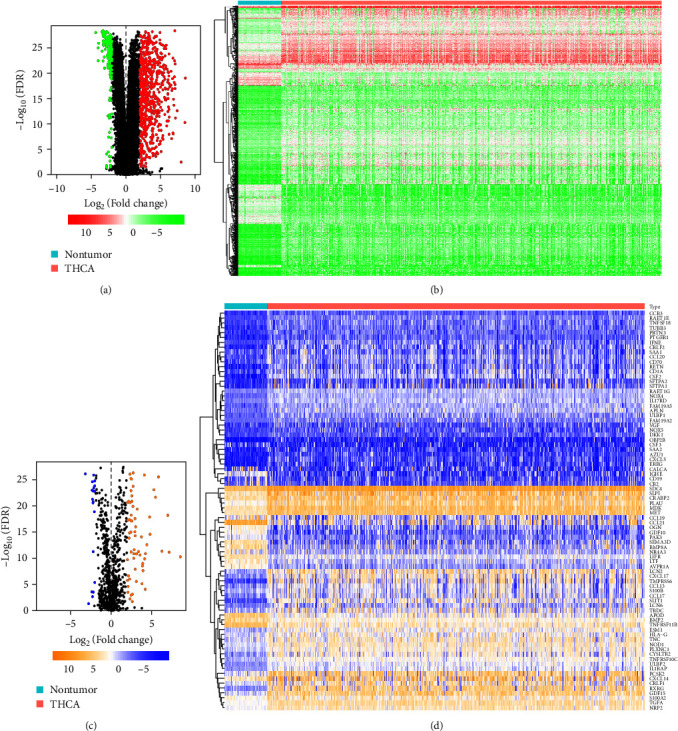
The DEGs and DEIRGs in The Cancer Genome Atlas. Volcano plot (a) and heatmap of (b) of DEGs between THCA and normal tissues. Volcano plot (c) and heatmap of (d) of DEIRGs between THCA and normal tissues. Green and blue dots denote down-regulated genes, red and yellow dots denote up-regulated genes. DEGs, differentially expressed genes; DEIRGs, differentially expressed immune-related genes; FDR, false discovery rate; THCA, thyroid cancer.

A total of 878 genes (676 upregulated and 202 downregulated) were considered as differentially expressed genes in THCA tissue samples compared with adjacent normal tissue samples (Figures 1(a) and 1(b)). We extracted 82 IRGs from this set of genes, which included 19 downregulated and 63 upregulated genes (Figures 1(c) and 1(d)).

### 3.2. PPI Network Construction and Function Annotation for Screened DEIRGs

**Figure 2:**
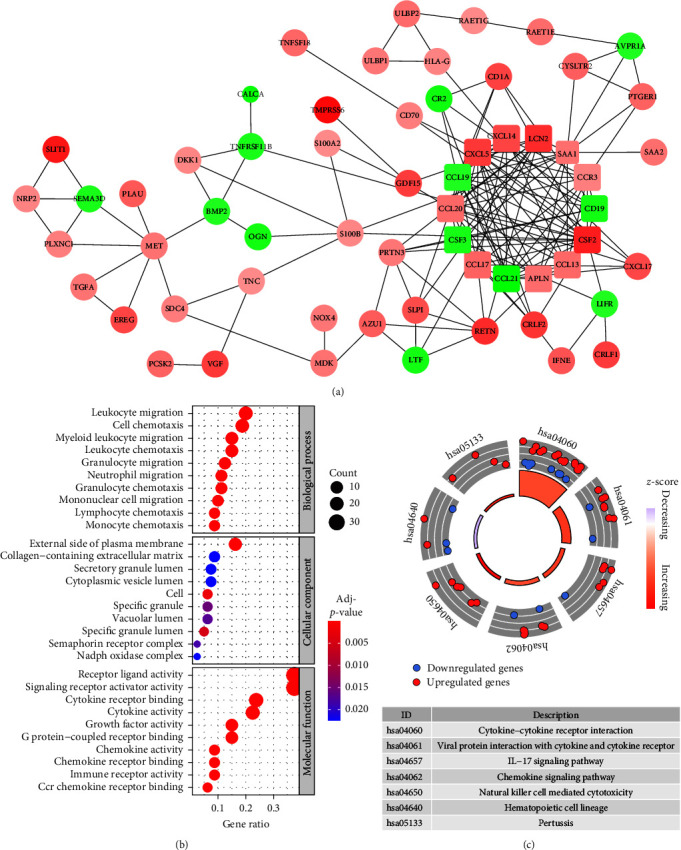
PPI network construction and gene functional enrichment of 82 DEIRGs. (a) PPI network. Red and green nodes represent up-regulated and down-regulated DEIRGs, respectively, and the color depth is positive related with |log2FoldChange|. The width of lines is based on combined score of protein interaction. The size of nodes is negatively correlated with *p*-value. Square nodes denote hub genes which interacted with more than eight proteins. (b) The top 10 significant biolgical process, cellular component, and molecular function that all DEIRGs involved in according to Gene Ontology analysis. (c) All significant KEGG pathways. DEIRGs, differentially expressed immune-related genes; PPI, protein-protein interaction.

We analyzed the interaction of all DEIRGs and visualized it in Figure 2(a). Gene functional enrichment was performed on all DEIRGs. Gene function enrichment analysis showed that “leukocyte migration” was the most relevant biological process, the cell components that most DEIRGs constituted were “external side of the plasma membrane,” and “receptor ligand activity” was the most significant molecular function that DEIRGs involved in (Figure 2(b)). The analysis of enriched pathways of DEIRGs showed that “cytokine-cytokine receptor interaction” was most common enriched pathway (Figure 2(c)).

### 3.3. Identification of DEIRGs with Prognosis Value in THCA

**Figure 3:**
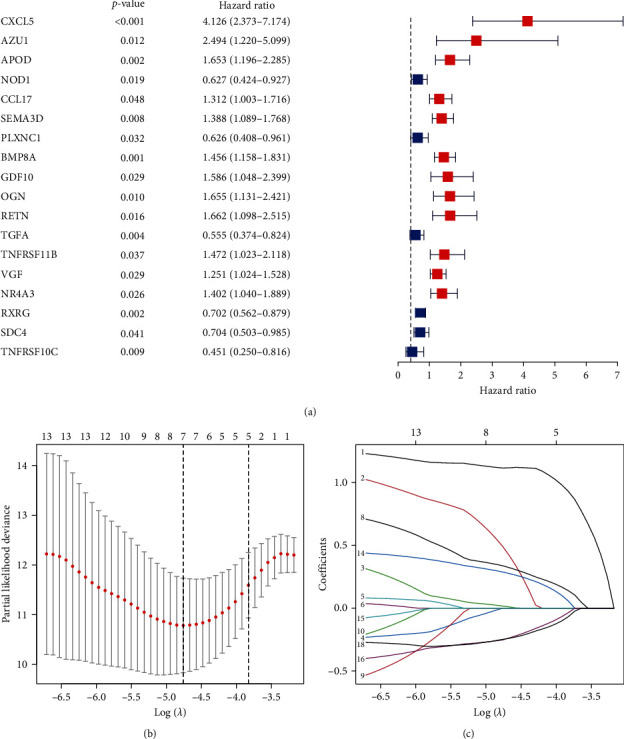
The process of the prognostic risk model establishment. (a) Forest plot of hazard ratios showing the survival-associated DEIRGs identified by univariate Cox analysis. (b-, c) The establishment of the immune-related prognostic model based on LASSO regression analysis. Seven DEIRGs with the highest prognostic values in TCGA cohort were identified. DEIRGs, differentially expressed immune-related genes; LASSO, least absolute shrinkage and selection operator; TCGA, The Cancer Genome Atlas.

The 487 THCA patients with follow-up of >90 days in the TCGA cohort were adopted as training cohort. The univariate Cox regression analysis was performed on all DEIRGs to evaluate the prognostic value of all DEIRGs for THCA patients from the TCGA training cohort. There are 18 DEIRGs were identified as prognostic DEIRGs which significantly correlated with OS in THCA patients (*p* < 0.05), 12 of them were prognostic risk factors (HR > 1) and the rest six were prognostic protective factors (HR < 1; Figure 3(a)).

### 3.4. Establishment of Immune-Related Risk Signature for Risk Scoring and Survival Prediction

To avoid overfitting and improve robustness, 18 prognostic DEIRGs which screened from univariate Cox analysis were included in the further subsequent LASSO regression analysis. Finally, seven DEIRGs (CXCL5, AZU1, APOD, NOD1, BMP8A, TGFA, and RXRG) were screened out as independent prognostic DEIRGs and were applied to construct the immune-related risk model (Figures 3(b) and 3(c)). The list of seven risk genes and calculation coefficients are shown in Table 1. We calculated each patient's risk score with the immune-related risk model in two separate cohorts: the training cohort from TCGA and the independent cohort from ICGC.

### 3.5. Validation of the Prognostic Value of the Risk Signature in the TCGA Cohort and ICGC Cohort, Respectively

**Figure 4:**
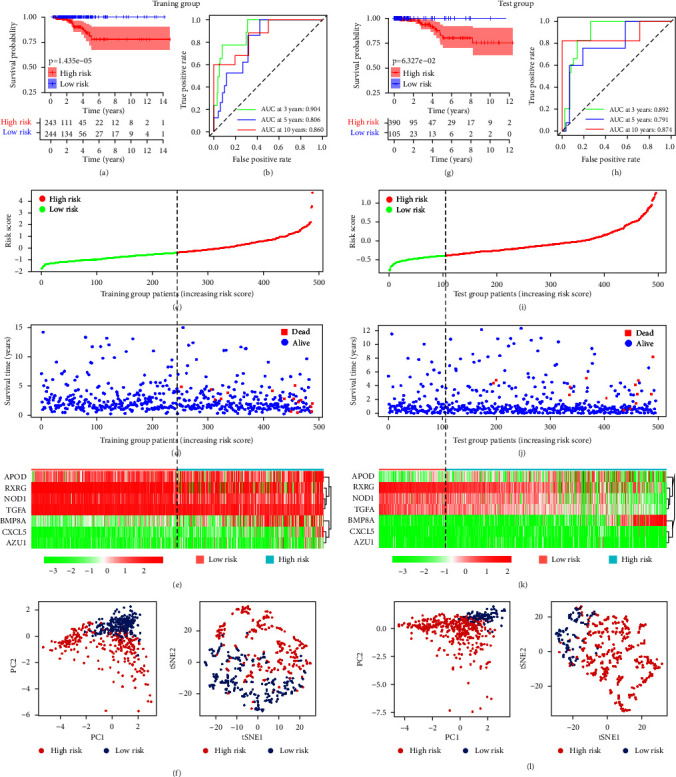
Internal and external verification of the immune-related prognostic model in the TCGA and ICGC cohorts, respectively. (a) Kaplan–Meier survival analysis of THCA patients in different risk groups in the TCGA cohort. (b) Time-dependent ROC analysis of the risk score model to predict the overall survival of THCA patients. (c, d) The distribution of risk scores, and corresponding survival status of THCA patients in the TCGA cohort. (e) Heatmap of seven risk genes that formed the risk model in the TCGA cohort. (f) PCA plot and *t*-SNE analysis for the TCGA cohort. (g, h) Kaplan–Meier survival plot and time-dependent ROC analysis of the risk score model for evaluating the prognostic value of the risk model to predict the survival of THCA patients in the ICGC cohort. (i, j, k) The distribution of the risk scores, corresponding survival status of THCA patients, and expression patterns of seven risk genes in the ICGC cohort. (l) PCA plot and *t*-SNE analysis of the ICGC cohorts. The high-risk and low-risk groups displayed variant immune statuses. ICGC, International Cancer Genome Consortium; TCGA, The Cancer Genome Atlas; PCA, principal component analysis; ROC, receiver operating characteristic; THCA, thyroid cancer; *t*-SNE, *t*-distributed stochastic neighbor embedding.

The median risk score stratified 487 THCA patients in the TCGA cohort into high-risk (*n* = 243) and low-risk groups (*n* = 244). According to the survival curve, low-risk group patients had a higher survival probability than high-risk group patients (*p* < 0.001; Figure 4(a)). The AUC values of the risk model for predicting OS at 3, 5, and 10 years were 0.904, 0.806, and 0.860, respectively (Figure 4(b)). We sorted the risk scores of patients in the training cohort and analyzed their distribution, the death toll increased with the risk score increase (Figures 4(c) and 4(d)). The heatmap described the expression patterns of seven risk genes involved in the risk model in different risk groups (Figure 4(e)). The results of principal component analysis (PCA) and *t*-distributed stochastic neighbor embedding (*t*-SNE) analysis for the TCGA cohort revealed that patients in low-risk and high-risk groups were distributed in different directions (Figure 4(f)), it instructed that our risk model could accurately risk stratification of TCGA patients depending on the variant immune status of patients.

In the ICGC cohort, the median risk score of the training cohort was still used as a critical value, and 495 THCA patients were stratified into a low-risk group (*n* = 105) and a high-risk group (*n* = 390). The survival probability of the high-risk group and low-risk group was compared and the result was consistent with the TCGA cohort, the survival rate of high-risk group patients was significantly lower in the ICGC cohort (Figure 4(g)). The AUC values of the risk score model for predicting OS of THCA patients from the ICGC cohort at 3, 5, and 10 years were 0.892, 0.791, and 0.874, respectively (Figure 4(h)). Figure 4(i)–4(k) showed the death toll of THCA patients increased with the risk score increase. The results of PCA and *t*-SNE analysis in ICGC cohort were in accordance with the TCGA cohort, patients in different risk groups in the ICGC cohort were also distributed in discrete directions (Figure 4(l)).

The results of internal and external validation demonstrated the general applicability and high stability of the immune-related prognostic model for predicting the prognosis of THCA patients.

### 3.6. Nomogram and Clinical Correlation Analysis of the Prognostic Risk Model

**Figure 5:**
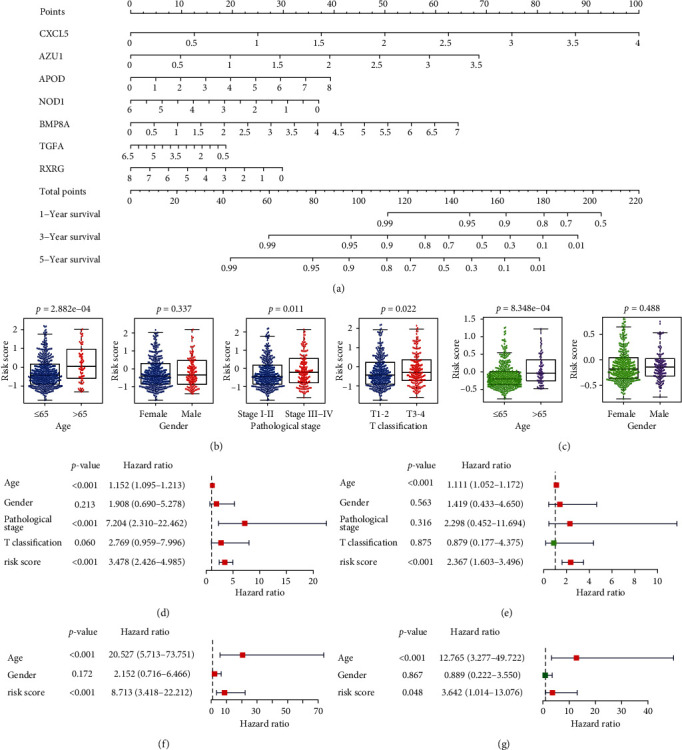
Nomogram and clinical correlation analysis of the prognostic risk model. (a) The nomogram of the immune-related risk model in the TCGA cohort. (b, c) Relationships between the risk score and the clinicopathological indicators of THCA patients in the TCGA cohort and ICGC cohorts, respectively. (d–g) Univariate and multivariate cox analyses of the risk score and clinical features of THCA patients in the TCGA cohort (d, e) and ICGC cohorts (f, g), respectively. The risk score model can be utilized as an independent prognostic indicator. AUC, areas under the curve; ICGC, International Cancer Genome Consortium; TCGA, The Cancer Genome Atlas; THCA, thyroid cancer; *t*-SNE, *t*-distributed stochastic neighbor embedding.

For clinical use, a prognostic model nomogram was illustrated according to the TCGA cohort (Figure 5(a)). To assess the clinical relevance and significance of the risk model, we analyzed the correlation between risk scores and clinical characteristics in the TCGA cohort and ICGC cohort separately. In the TCGA cohort, the prognosis model was significantly correlated with age, pathological stage, and T classification (Figure 5(b)). In the ICGC cohort, the clinical message of THCA patients is limited, the prognosis model was also significantly correlated with age (Figure 5(c)).

Then, univariate and multivariate Cox regression analyses were conducted on risk score and clinicopathological indicators to evaluate whether the risk score model was an independent prognostic indicator independent of other clinical parameters in THCA patients. According to the results of univariate Cox analysis, in the TCGA cohort, there was a significant positive correlation between age, pathological stage, risk score, and prognosis of THCA patients (Figure 5(d)). The results of multivariate Cox analysis prompted that age and risk score were independent prognostic indicators of THCA patients (Figure 5(e)). In the ICGC cohort, age and risk score were significantly associated with survival (Figures 5(f) and 5(g)). All results confirmed that the immune-related risk model can be applied independently for the prognosis prediction of THCA patients.

### 3.7. Potential Roles of Immune Infiltrating Cells in Prognostic Prediction

**Figure 6:**
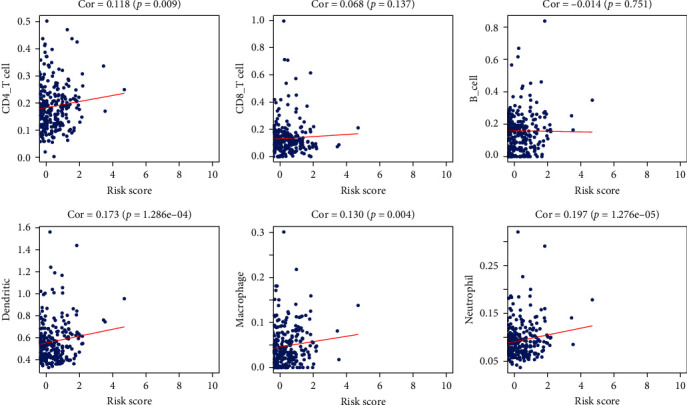
Association between the prognostic model and infiltration proportion and abundances of six types of immune cells.

To determine whether the prognostic risk model can reflect the condition of the tumor immune microenvironment of THCA patients, we did a correlation analysis. The association between the scores of CD4+ T cells, macrophages, dendritic cells, neutrophils, and risk score were significant and positive, suggesting an association between increased infiltration and risk score (Figure 6).

### 3.8. Function Analysis for Seven Risk Genes

**Figure 7:**
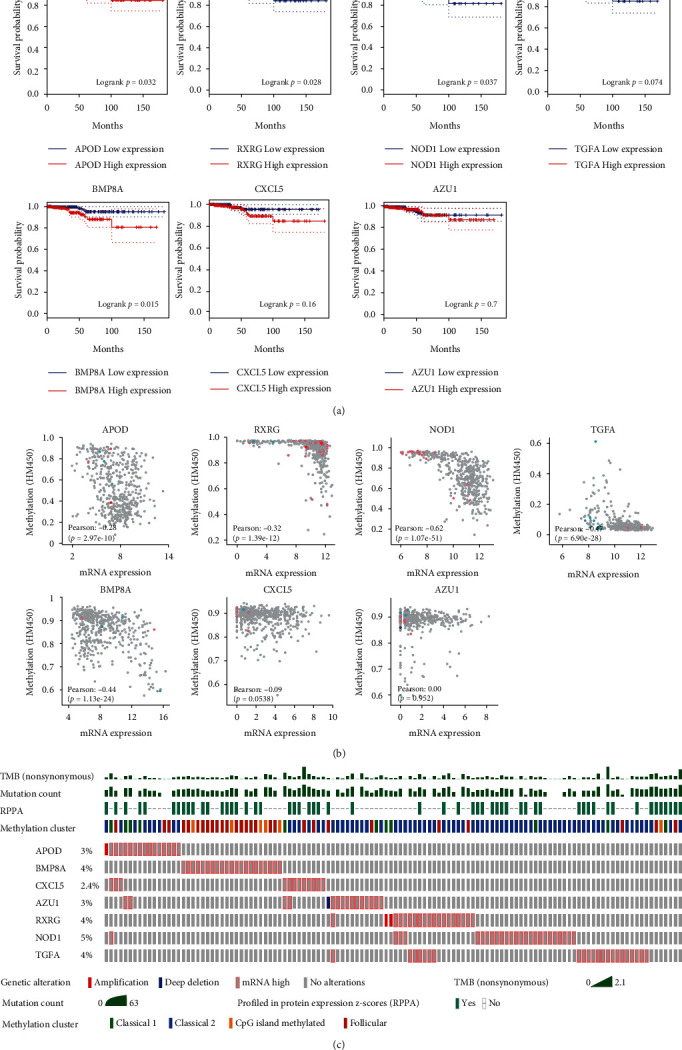
Prognostic value of seven risk genes in THCA. (a) Survival correlation analysis between expression of seven risk genes and overall survival of THCA patients. (b) Relationship between expression of seven risk genes and methylation levels. (c) Genetic alteration of seven risk genes and corresponding message of TMB, protein abundance, and methylation cluster in THCA cases. TMB, tumor mutation burden; THCA, thyroid cancer.

Previous bioinformatics analyses showed that APOD, BMP8A, CXCL5, and AZU1 were high-risk genes and RXRG, NOD1, and TGFA were low-risk genes. To investigate the association of seven risk gene expressions with the prognosis of THCA patients, survival curves were plotted. As shown in Figure 7(a), high-risk genes were related to poor prognosis, and low-risk genes were related to longer survival. In Figure 7(b), the correlation between mRNA expression and methylation levels of the seven genes was exhibited. We also analyzed and visualized genomic alterations of the screened risk DEIRGs, and the corresponding message of TMB, protein abundance, and methylation cluster in different THCA cases (Figure 7(c)).

## 4. Discussion

THCA is a high-incidence malignant endocrine cancer [14], though the disease-related mortality of THCA is relatively low, it is worth noting that many patients suffered repetitive treatments due to the recurrence of THCA, at the same time the clinical burden increased [15, 16]. So, to identify more aggressive THCA and predict the recurrence of THCA effectively, the risk stratification system was proposed by the American Thyroid Association (ATA) guidelines in 2016 [17]. The TNM staging system is one of the major players in the ATA risk level classification.

TNM staging system is the reference standard for THCA prognosis predicting [18, 19], a significant function of the staging systems is to help to make the optimum therapeutic method in the clinic [20], hence it is useful and recommended for all THCA patients [17]. With the accumulation of survival data and a deeper understanding of tumor behavior and clinical outcomes, the staging model of THCA is always updating. In fact, several clinicopathological indicators and molecular findings that are not included in the TNM staging system are considered in the ATA risk classification [21]. In the current study, we want to explore conceivable molecular prognostic markers that can incorporate into the risk classification system.

The immune system plays a critical role in THCA progression [22]. There is accumulating evidence confirming that infiltration of immune cells in THCA can predict prognosis [23, 24]. Some researchers deemed that immune phenotype should be taken into account as a parameter for improving the management of THCA patients and attempt to define a new classification of THCA according to immune signature making use of genomic and transcriptomic analyses [25]. Thorsson et al. [26] identified six immune subtypes for over 10,000 cancers of 33 different types through a huge immunogenomic analysis using TCGA data in 2019. Some studies considered the Immunoscore to be exploited in the thyroid and found that immunoscore has a negative correlation with thyroid differentiation score [27]. Thus, increasing awareness of the changes in immune function involved in the development of THCA could benefit prognosis prediction and precise personalized therapy. In this study, we attempt to refine the prognostic risk model of THCA by incorporating immune-related molecular prognostic markers into the prognostic system to enhance the prognostic accuracy of clinical outcomes prediction and benefit to targeted therapy.

In recent years, precision genomic medicine is prevailing. To screen out specific and stable predictive factors for survival prognosis from the immense amount of medical data sets with complete clinical outcomes is the top priority of precision genomic medicine [28]. We explored immune-related prognostic factors using bioinformatics analysis based on precision genomic medicine in the current study. Our study constructed a novel immune-related prognostic risk model for THCA patients first and validated it in the TCGA cohort and ICGC cohort, the risk model had general applicability and high stability to predict the prognosis of THCA patients. Of course, our study has several limitations. On the one hand, the lack of information of prognostic information on local THCA patients' cohorts to further tested our results. On the other hand, our findings were obtained by pure bioinformatics analysis, so the function of hub genes and prospective medicines is required to be further confirmed by scientific investigation in vitro and in vivo, further studies on seven DEIRGs composed of the prognostic model are needed.

## 5. Conclusions

We explored the immune mechanism of THCA and established a reliable prognostic evaluation system using seven DEIRGs as risk genes, the risk score calculated according to our model can be used as an independent prognostic indicator. Our study may benefit to enrich the therapeutic targets of THCA. Moreover, our risk model can reflect the infiltration of immune cells in THCA patients and will benefit in predicting the sensitivity to immunotherapy of patients.

## Figures and Tables

**Table 1:** Coefficients of seven independent key prognostic immune-related genes (IRGs).

IRGs	Coefficients
*CXCL5*	1.06145781903057
*AZU1*	0.411650303964923
*APOD*	0.123387784068972
*NOD1*	−0.0368064508535378
*BMP8A*	0.327117704344495
*TGFA*	−0.138681433742042
*RXRG*	−0.159939305539482

## Data Availability

The raw data supporting the conclusions of this manuscript will be made available by the corresponding author, without undue reservation, to any qualified researcher.

## Abbreviations

AUC: Area under the curve
DEIRGs: Differentially expressed Immune-related genes
GO: Gene Ontology
ICGC: International Cancer Genome Consortium
IRGs: Immune-related genes
KEGG: Kyoto Encyclopedia of Genes and Genomes
KM plotter: Kaplan–Meier plotter
LASSO: Least absolute shrinkage and selection operator
Log_2_FC: Logarithm of Fold Change/Log2Fold Change
OS: Overall survival
PPI: Protein-protein interaction
TCGA: The Cancer Genome Atlas
THCA: Thyroid Cancer.

## Supplementary Materials

Supplementary Materials The Supplementary Figure 1 is a flowchart developed to fully describe our study. Figure S1: Overall flowchart of this study.
