# Supplementary figures and images for: Mining Prognostic Biomarkers of Thyroid Cancer Patients Based on the Immune-Related Genes and Development of a Reliable Prognostic Risk Model
Source: Mediators Inflamm. 2023 Jul 31;2023:6503476. doi: 10.1155/2023/6503476 (PMC10406562; doi:10.1155/2023/6503476)

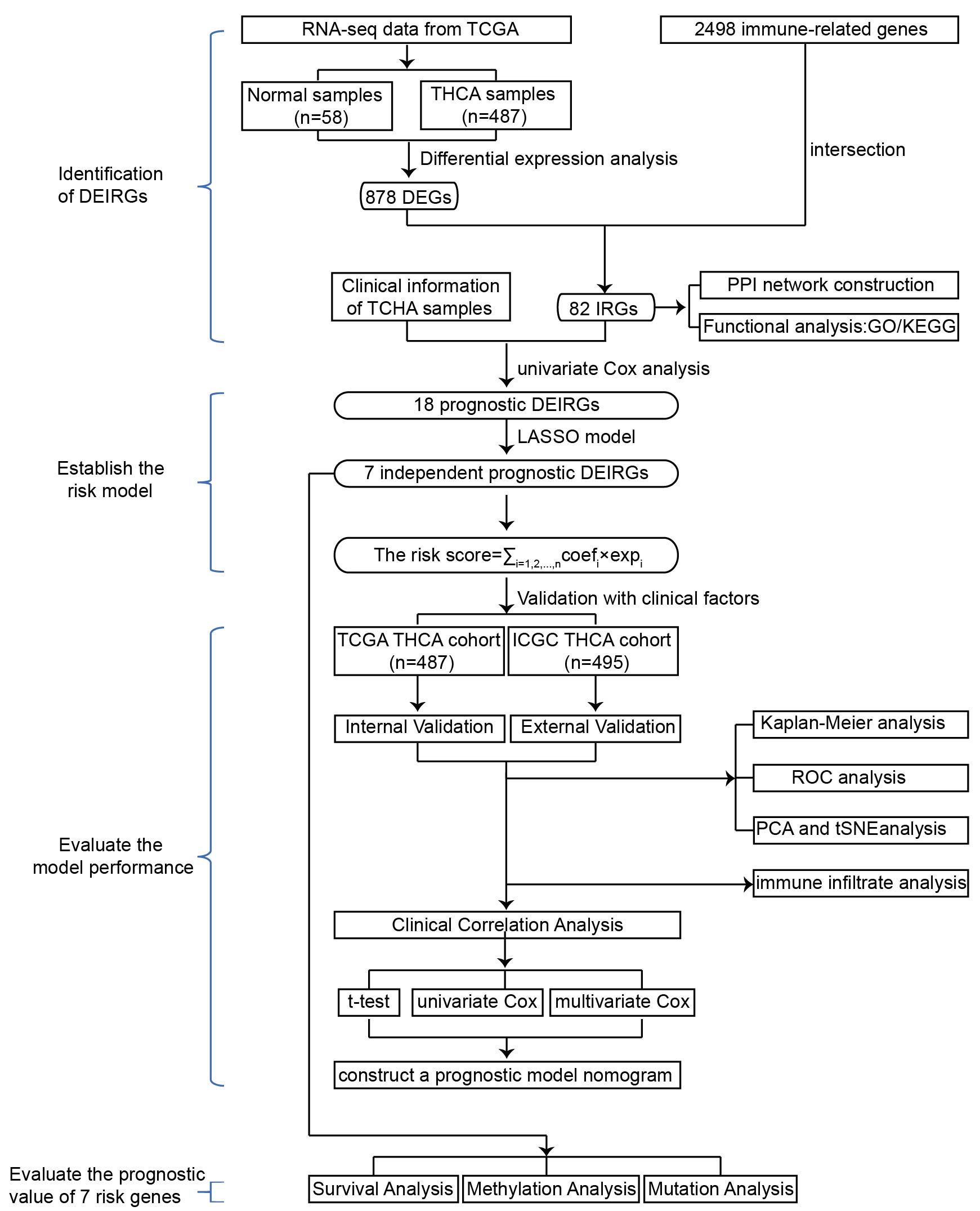

Supplement: Supplementary Materials — The Supplementary Figure 1 is a flowchart developed to fully describe our study. Figure S1: Overall flowchart of this study. [file 6503476.f1.jpg]
